# The geography of abortion: Discourse, spatiality and mobility

**DOI:** 10.1177/03091325221128885

**Published:** 2022-09-25

**Authors:** Sydney Calkin, Cordelia Freeman, Francesca Moore

**Affiliations:** School of Geography, 4617 Queen Mary University of London, London, UK; Department of Geography, 3286 University of Exeter, Exeter, UK; Homerton College and Department of Geography, 2152 University of Cambridge, Cambridge, Cambridgeshire, UK

**Keywords:** abortion, discourse, spatiality, mobility, power

## Abstract

Abortion has historically been ignored in geography. Although bodies and pregnancy have been increasingly studied since the 1990s, a reticence around abortion remains. In recent years, however, this has begun to change. This article critically reviews how geographers and other scholars are now considering abortion and uses three conceptual lenses of discourse, spatiality and mobility to argue that abortion should be a mainstream topic of critical concern for geographers. Through these themes we show that geographical attention to abortion makes questions of space, power, and citizenship visible in new ways and, furthermore, in ways that are only recently possible.

## I Introduction

Abortion, or the termination of pregnancy, is a geographical issue that has been hitherto marginalized within the discipline. As a private, often criminalized, procedure which provokes reactions ranging from support to condemnation and even incarceration, abortion is a very challenging research topic. For example, Moore (2010) reported significant barriers to researching illegal abortion in the 2000s as a graduate student due to the perceived controversy around the topic. Always timely, always controversial, often in the news – most recently with the 2022 overturning of Roe versus Wade – abortion merits sustained scholarly attention by geographers. In this paper, we show why abortion is such an important topic for geographical analysis and how the conversation around it, both academically and culturally, may be changing. We argue not only that it should be studied in its own right but we also show the ways in which the study of abortion offers an important new entry point to larger questions, including the relationship between the spatial and the political in questions of statehood, citizenship and power. One of the most significant results of an approach using abortion as a lens on broader geographical topics is that it reveals linkages through time and space which go otherwise unseen.

Interest in pregnancy entered the discipline through scholarship on pregnant embodiment and the experiences of pregnant women in public spaces. Longhurst’s (2000) work was foundational to this area of work, because she demonstrated the importance of the body – ‘the geography closest in’ – as a legitimate site of geographical enquiry. This bodily focus is a relatively new endeavour for geography, with Mountz (2018) and Jeffrey (2020) showing separately the ways in which geographical scholarship has ‘caught up’ and expanded its analytical reach with the turn to the body. Jeffrey (2020) explicates the type of work being done, noting that it is both the discursive conceptualisation of the body *and* the fleshy reality that are of interest to geographers. This new work clearly engages with Katz’s call for feminists to undertake study of the ‘messy fleshy stuff of everyday life’ (Katz, 2001:711), perhaps bringing greater prominence to feminist scholarship in geography which has, as Domosh and Morin (2003) observed, rarely travelled under its own name. Therefore, through work on the body, pregnancy has become a topic of appropriate concern for geographers.

Work on reproductive geography is flourishing across the discipline. Colls and Fannin (2013) and Lewis (2018), for instance, have developed the study of bodily interiority through the investigation of uterine and placental geographies. Others have examined geographies of pregnancy (Mansfield, 2012; Woliver, 2010), childbirth and rearing (Boyer, 2018; England, 1996; McKinnon, 2016), and pregnancy loss (McNiven, 2016) alongside discussion of the ethical issues raised by researching these sensitive topics (Moore, 2010). Schurr (2018) situated reproductive bodies and consumers of reproductive healthcare in neoliberal globalisation, observing the highly intricate networking of bodies, services, and capital in a global marketplace of reproductive healthcare. Social science work on assisted reproductive technology has been mainly within anthropology, sociology and law, although this work illustrates a rich geography. For example, work by Ivry (2010) and Lupton (2013) consider how ultrasonographic images take on different medical and moral meanings in different cultural contexts. Gurtin’s (2011) piece on the extra-territoriality of law which criminalized Turkish citizen’s accessing third-party assisted reproduction in other nation-states echoes Freeman’s (2017) work on the elasticity of law in relation to reproductive bodies.

There is a growing body of scholarship under the umbrella of ‘reproductive geographies’ but this work predominantly focuses on fertility, pregnancy and birth. The important book *Reproductive Geographies* (England et al., 2018), edited by Marcia England, Maria Fannin and Helen Hazen, for example, includes work on artificial insemination, commercial surrogacy and birth experiences across its eleven chapters, but never directly deals with abortion. Although the cultural turn centred pregnancy and pregnant embodiment as legitimate sites of geographical enquiry, abortion has, until very recently, been notably absent from this literature. Where geographers have engaged with abortion, it has generally been through the lens of legal and population geography (Brickell and Cuomo, 2019; Tyner, 2015). Abortion is also discussed to illustrate arguments about politics and protest (see e.g. Mitchell’s 2005 work on ‘SUV’ models of citizenship which takes abortion clinic protests as a key site of analysis), but has rarely been studied as a topic in its own right. The embodied experience of desiring, attempting, failing or succeeding in ending a pregnancy has not often been the subject of study in geography. Yet, this is changing. Several papers on the topic have been published each year in geography since 2018. This recent work shows interest in cross-border abortion travel, though much of this work (with a few exceptions) comes from historians and sociologists (see Stettner et al., 2017; Sethna and Davis, 2019; Side, 2020; Gilmartin and White, 2011; Baird and Millar 2020).

Recent intellectual developments in geographical scholarship set the scene for a more sustained and multi-faceted engagement with the topic of abortion within the discipline. There are two reasons for this: an increasing attention to scale and a renewed focus on the onto-epistemology of the discipline itself, both of which promise a different kind of geography. The question of scale has long fascinated geographers and its analyses are becoming increasingly political. In 2000, Marston noted that investigations of scale must include social reproduction (Marston, 2000). The importance of the scale of the everyday and embodied experience is increasingly seen across geography, especially in scholarship on feminist political geography (Brickell and Cuomo, 2019; Coddington, 2021) economic geography (Yarker, 2017) and austerity (Strong, 2020). This research has most recently given rise to remarkable ‘autocorporeal’ research methods where Strong (2022) employed his ‘tasting body’ to scrutinise forms of embodied privilege in his investigation of food bank diets. Turning to questions of onto-epistemology and the possibility of changes in the discipline, two recent papers from Oswin (2020) and Kinkaid et al. (2021) reactivated and expanded questions that feminist geographer Gillian Rose asked of geographical scholarship in 1995. Rose (1995) raised questions about the kind of knowledge that is possible in a subject whose DNA is the privileged white male explorer. Oswin (2020) critiqued geography’s white supremacist heteropatriarchal grounding and observed a turn away from geography’s mainstream scholarship. Similarly, Kinkaid et al. (2021) observed a new and transformational dialogue on power, specifically in relation to whiteness, masculinity and cisheternormativity. This scholarship attests to the fact that the discipline has reached a critical juncture where the types of scholarship carried out under the sign of geography are changing in fundamental ways.

The historical absence of abortion from geographical scholarship – or its treatment as a peripheral topic – matters for at least four reasons. First, abortion is a common experience and is one among many reproductive events across a person’s life course. Globally, the Guttmacher Institute estimates that 73 million abortions occur every year (2018). Abortion may be shrouded in silence and stigma, but it is not rare. Second, the absence of abortion scholarship in the discipline matters because it reinforces and contributes to the stigmatisation of abortion in society at large. As geographers who study abortion, we have all become accustomed to this kind of response to our work – ‘oh god, abortion, how depressing!’. Historically, the academic silence around the topic suggests that it has not been seen as a respectable or appropriate topic for scholarly analysis. So much about sex and sexuality is considered private, and this has undoubtedly exerted an influence in intellectual life. This reaction to the idea of abortion reflects its status as a political issue that is always being contested, which is the third reason its absence from geography matters. Although abortion regulations differ dramatically between countries, it remains a controversial, ever-changing and highly politicized topic everywhere. Geographers of health and medicine have richly illustrated the way that socio-spatial inequalities impact health and how inequalities in health shape social space in turn (Bambra, 2016). Unevenness in the reproductive healthcare landscape exacerbates inequalities along the lines of race, gender, sex and ability (Ross and Solinger, 2017). Fourth, and finally, we argue that the absence of abortion from geographical scholarship is a gap that must be addressed because the issue of abortion is uniquely positioned to illustrate the relationship between state and society. It stands alone as an essential healthcare procedure that is often also a criminal act, and therefore as a useful lens through which we can understand the political power structures that act upon reproductive (and non-reproductive) bodies. This paper argues that abortion should be placed at the centre of a geographical analysis to garner new perspectives on key topics of social and political enquiry.

The paper proceeds by using three key themes – discourse, spatiality and mobility – to demonstrate the significance of abortion as a topic in its own right and to sketch out ways in which it can offer new insights into broader themes within geography.

1. Discourse: A thematic approach to abortion discourse highlights the ways in which exploring both the narration of the procedure and its representations through time should be central to a research agenda on abortion. Both of these foci offer a way to connect the procedure to larger scale political questions.
2. Spatiality: The spaces in which abortion is regulated speak to wider gendered norms and structures of governance that regulate women’s lives. Abortion is frequently understood as a site of heated public debate, but the private politics of abortion remain poorly understood, especially clandestine abortion practices.
3. Mobility: Abortion is fundamentally about mobility because, across scales, from the clinic to the nation-state, bodies, pills and knowledge are on the move in ways that reflect, reinforce and contest power relations. Abortion mobilities encompass barriers to movement, the privilege of not moving, and technologies that facilitate (im)mobility.

## II A discursive approach to abortion

One of the hallmarks of geographical enquiry is an attention to geometries of power. To do this type of analysis is often to take discourse as a starting point. In Foucauldian terms, discourse is a set of ideas or way of thinking about the world. The context of ideas is important, as Foucault (2001) noted; the conditions under which it is possible for things to be thought are key to our understanding. In this section, we suggest two main approaches to researching abortion discursively, using the concepts of narration and representation, and we explain how these contribute to an abortion research agenda.

Approaching abortion discursively opens up key analytic space that prevents the bracketing of the issue as a private, women’s matter which artificially narrows the relevance of abortion to wider social and political issues. Firstly, by exploring representations of abortion we can take apart the assumptions at work to expose the social and cultural ideas that underpin and drive both the regulation of abortion and contestations over the law. This includes excavating norms about how women should behave and their links to wider societal goals about family and population through time. Secondly, the narration approach to abortion discourse offers a way to engage with the material reality of the procedure through case studies and testimonies and also affect. This facilitates engagement with the everyday experience of abortion politics and has the scope to include matters such as religion. Both of these discursive themes are highly geographic, drawing attention to spatiality, uncovering the ways in which abortion is inherently geographical in its relevance to a variety of different scales and spaces, and, crucially, the connections between these.

### 1 Narration: Exploring testimony, affect and lived experience

The latest scholarship on abortion reveals the importance of engaging with everyday experiential discourses of abortion involving political, emotional and moral articulations of the issue. This, of course, chimes with the broader trend towards the everyday, bodily and affective across geography described earlier. O’Shaughnessy’s (2021) narrative analysis of Ireland’s campaign for abortion rights focuses on the implications of the conservative political approach of the pro-choice campaign which, she argues, has meant that Ireland has not yet been successful in achieving the destigmatisation of abortion. Paradoxically, this narrative failure sits alongside the success of Repeal. This is a clear indication of the ways in which the narration and conceptualisation of abortion politics have consequences in the everyday.

Similarly, Thomsen et al.’s (2022) work on mobile crisis pregnancy centres in the USA implicates the units in the spread of Evangelicalism, medical misinformation and anti-abortion ideology using unprecedented techniques. Moreover, the centres exist in a legal blind spot enabling them to evade regulation. Thomsen et al. (2022) suggest that these ‘unruly’ organisations are now the front line of the anti-abortion campaign because of the deftness of their tactics and their ungovernable physical mobility.

Tackling this new type of anti- abortion activism seems like a form of ‘whack-a-mole’ but might, Thomsen (2022) suggests, motivate action through outrage among people who are politically disenaged. Thomsen connects the unscrupulous practices of crisis pregnancy clinics to broader political issues of taxpayer funds, data privacy and public health. This re-scaling is yet another important demonstration of how the bracketing of abortion as a private, women’s issue slights other, very important perspectives that embed the issue in much broader questions of citizenship, law and politics.

### 2 Representations: A close reading of the past

There is considerable variation in the legal treatment of abortion around the world. Such a mosaic of regulatory approaches has already been connected to the social and cultural contexts of law-making including the maintenance of machismo conservative regimes in Chile (Freeman, 2017) and heteronormative, racialized and religious constructs of the family (Hanafin, 2007). Although the law is seen in some respects as an abstract system that gives a lack of attention to material structures that embody social relations (Gill et al., 2021), this extant scholarship implicates the legal regulation of pregnancy as a route to many larger-scale social and cultural goals. This nexus of individual and total clearly connects individual reproductive behaviour to wider questions of policy and power (Chen, 2003). This scalar politics suggests the importance of excavating the discursive understanding of abortion procedures across time and space. Seen in this way, then, abortion occupies a special position academically as a relatively under-studied entry point to the study of larger-scale political processes such as citizenship and gender roles and even religion. By placing abortion in a longer genealogy, we can also trace the influence of particular historical constructs of gender and norms about how women should behave into the present day.

Regulation creates particular legal subjectivities, or identities in the law, all of which are gendered, classed and racialized and, moreover, may contain echoes of the colonial past (Gregory, 2004; Khalili, 2013; Moore, 2018; Said, 1978). In the case of the regulation of abortion, the legal address of the pregnant body seems to bring into being a subjectivity that self-evidently needs regulation, or reformatory discipline. To trace the creation of this ‘problem population’ is a richly complex historical research endeavour that encompasses naming the influences and entanglements in the law and also, by extension, in geographical scholarship. This involves a reckoning with past academic thought as well as history itself. According to Chadwick (2021), this is a political process of researcher (and reader) discomfort which is central to ethical and accountable feminist research. To do this, then, in the geographical study of abortion, is to trace the history of the concept, in its regulation and its study by naming the processes and beliefs that have had an influence on its meaning. It is in this way that we can connect a routine medical procedure regulated by criminal law to wider spatial and political questions about nationhood, citizenship and colonial power across time.

In the UK, one of the most significant piece of legislation to regulation abortion was the 1861 Offences Against the Person Act which originated in the Victorian era. The social context of this legislation was that Victorian women’s primary social role was maternal. Their purpose at the turn of the 20th century was to rear the next generation in order to stave off Britain’s racial and geopolitical decline (Davin, 1978; Soloway, 1982). Any transgression from this norm was widely censured (Rosenman and Klaver, 2008) largely because it threatened established gender hierarchies and social norms. These powerful Victorian notions of ‘good motherhood’ and maternal martyrdom were enshrined in law which has given them a significant afterlife and a cultural stranglehold in the present (Moore, 2018). That colonial logics migrate through time is clear in the impressive reach and longevity across space and time of the 1861 Offences Against the Person Act. It remained in place in England and Wales until in 1967, The Abortion Act replaced the 1861 Act. However, the 1967 Act was a piece of legislation that did not grant women the right to end an unplanned pregnancy but gave doctors the discretion to decide whether there are medical grounds to support a woman’s request for abortion (Bristow, 2013). Here we see the nineteenth-century social control function of medicine as described by Oakley (1980) at work yet much later in time.

Moreover, scholars have also shown how prohibitive 19th century abortion laws were imposed on Caribbean colonies over a century ago but in later years modified only in Europe (Pheterson and Azize, 2005). Northern Ireland is another significant example of the endurance of the 1861 Act and its colonial logic. The 1861 Act remained in place in Northern Ireland until 2019 and abortion access there remains complex. The regulation of private, childbearing or reproductive behaviour by governmental or other authority has therefore become a longstanding norm. That is to say that the private decision-making behaviour of individuals and couples around childbearing has always, paradoxically, been available for public scrutiny in a *marbling* of private and public spheres (Brown, 2006). Riley (1988) has termed this process a form of ‘corrective inspection’. Furthermore, these very Victorian forms of regulation made a clear connection between women and childbearing, creating an important cultural signal about how women should behave. Legalisation of abortion would presumably uncouple the link between women and childbearing and thus threaten Britain’s imperial might. A genealogical approach to the 1861 Act would therefore characterize it as an artefact of the era of the ascendancy of the Victorian British white male middle-class and, by extension, colonialism. As Said (1978) has shown, the ‘Orient’ was a sexualized female realm, ripe for plunder and control. In Foucauldian terms, then, society’s ‘others’ such as women were always governed through those pre-disciplinary powers of the sovereign, the very violent forms of bodily regulation; and were populations deemed incapable of possessing self-managing power (Pierce and Rao, 2006).

### 3 Protecting women?

Despite the maternal ideal, 19th and early-twentieth century Britain saw birth rates fall steadily and abortion was an accepted part of working-class life (Moore, 2013). The government expressed concern at the number of abortions carried out each year and specifically feared that women were at the mercy of backstreet abortionists. A shadowy figure in history (Jones, 2011), the abortionist was considered by government to be a threat to women; at once dangerous and unscrupulous. Yet, this logic of protection, the construction of the abortionist as a terror upon society and the abortion-seeking woman as vulnerable and naïve, was a particular choice of those in power. Moore (2013) has shown that illegal abortionists could actually be much-loved healthcare practitioners that were embedded in the community, providing a range of services across a woman’s entire life course. Moreover, as case studies across space and time from present-day Chile, 1960s Chicago and early twentieth-century Britain show, women have been proactive and resilient in their self-interested negotiation of the law by accessing the procedure through trusted networks of female friends (Brown, 2006; Freeman, 2017; Moore, 2013) and by passing down knowledge through generations (Bush-Slimani, 1993; Monchalin, 2021). That is not to say that women are not at risk of exploitation or harm (Freeman, 2017), but it is, however, to say that the discursive *framing* of the abortion-seeking woman as a criminal and the object of concern facilitates a particular type of social regulation which served goals of population control. Governance of abortion enabled the disruption of longstanding social norms about female conduct which formed the basis of many societies. The discursive framing of women as in need of protection was also located by Calkin (2020) who argues that the 2018 Repeal campaign to legalize abortion in the Republic of Ireland was characterized by repeated narratives about young, vulnerable, needy abortion-pill users.

Similarly, in the March 2020 COVID-19 lockdown in the UK, the government prevaricated over whether women should be allowed to take abortion pills at home. It gave temporary approval for patients to take both doses of early medical abortion at home without an in-person clinic visit. The government then made a U-turn before reversing its decision. The U-turn indicates some reluctance to grant women the autonomy of self-administered abortion at home and to do so would clearly disrupt what Wainwright (2003) has termed the ‘constant medical supervision’ of reproductive bodies. There was a similar moral panic years earlier over the de-regulation of the morning after pill and its subsequent availability over-the-counter in pharmacies. The colonial logic of an unruly population in need of reformatory discipline is clearly at work here. The governmental logic of women as in danger brings into being a population that self-evidently required the protection and oversight of the law *and* the doctor. As Kearns (2009) has shown, it is important to excavate the priorities exercised in the advancement and influence of a particular form of knowledge to which there clearly were alternatives.

A discursive approach to abortion highlights the historical assumptions about gender and social roles and the important cultural and regulatory work they do in abortion politics right into the present. Examples including the longevity of the 1861 Act across the globe and governmental prevarication about allowing abortion at home in pandemic times indicate not only the stranglehold of Victorian logics of care and gender, but also how easy and persuasive they are to deploy. Closely related to this is the spatiality of abortion as a private procedure with significant public interest that simultaneously occupies different spaces and scales. This is explored in the next section.

## III The spatiality of abortion

Abortion can be studied at multiple scales: it is at once an embodied experience, a medical procedure, an extensively regulated and litigated set of legal regulations, and a frequently debated political topic. We might study abortion’s spatiality in the numerous socio-legal settings including of the womb, the body, the clinic, the hospital, the state, the region and so on. The spaces in which abortion is regulated speak to wider gendered norms and structures of governance that regulate people’s lives, historicised above. To theorise abortion’s spatiality, this section starts by exploring the links between territory and abortion regulation. It then discusses the function of abortion restrictions in wider structures for governing population size, population health, national identity and symbolic borders of the territory. It concludes by showing how abortion is governed through the delegation of national abortion law to medical practitioners, by which clinical spaces become sites to monitor and manage pregnancies.

### 1 Territorializing abortion governance

To theorize abortion’s spatiality, we begin with a brief illustration. In 1966, after a decade of steadily falling birth rates, Romania sought to boost its population by banning abortiton except in a few limited circumstances. Its enforcement enrolled a variety of geographical scales and public institutions in the work of surveillance, all in service of broader population-level fertility goals. At one level, abortion was understood as a medical procedure that could be prevented if the patient could not access the clinical space, the clinical equipment, or the abortion provider all at once. As a result, hospitals were extensively monitored to prevent illicit abortions. In a system called ‘Territorialization’, only a few hospitals in each region were permitted to perform abortions or provide care after miscarriage (Kligman 1998: 63). The chief obstetrician/gynaecologist of each designated hospital was tasked with the unit’s achievement of reproductive targets, as well as personal responsibility for approving treatment of every patient. The surgical equipment needed for abortion was controlled by a different designated member of staff, who recorded every doctor who requested the equipment (and their patient). Failing to return abortion surgical equipment was understood as intent to perform illegal abortions (Ibid. 63).

Hospital surveillance only went so far: geographic and demographic data were also used detect illegal abortions. By comparing statistical data in each region, officials compared the population of women of child-bearing age, the number of recorded pregnancies, and the number of women who presented at hospital with suspected abortions. They sought to identify ‘discrepancies’ between expected and actual birth rates that might signal the occurrence of abortion. Places with low birth rates and high prevalence of women presenting at hospitals with miscarriages were labelled as ‘zones at risk’ and within these zones, medical professionals’ were individually scrutinized for illicit income derived from abortions (Ibid. 99).

Romania’s abortion ban is a notorious case, but it has wider insights for the geography of abortion because it illustrates the ‘studied combination of vertically and horizontally interwoven sanctions, suspicions, fears and enticements’ through which population-level governance is enacted through abortion regulations at the individual level, in spaces like workplaces, hospitals and local government (Kligman 1998: 61). Analogous mechanisms of abortion regulation can be found across the world, all of which rely on control of individual pregnancies and pregnant people to enact wider biopolitical aims. To name just one: in June 2022, the Polish Minister of Health signed an order requiring doctors to register the pregnancies of all their patients on the national medical record database, raising fears that this data could be used against abortion-seekers in the country (Koschalka, 2022). Below, we explore two spaces in which these mechanisms operate: the political and symbolic borders of the nation-state and the socio-legal boundaries of the clinic.

### 2 Abortion and natalist policies

Political projects of nation-building and boundary-drawing are intimately intertwined with questions of population size and composition. The management and reinforcement of the social order often takes place through interventions to control and monitor women’s sexuality: at a symbolic level, the sexuality of girls and women is often conflated with national identity and its boundaries. At an embodied level, women’s biological capacity to reproduce the state’s population generates management and surveillance tactics (Collins, 1998; Petchesky, 1984; Yuval Davis, 1997). In biopolitical terms, reproductive bodies occupy an important ‘threshold’ between the individual and national population (Deutscher, 2010). In this regard, women have historically been understood as both *subjects* of this governance and *spaces* for governmental action. Racialization and racism are central to understanding the politics of pro- and anti-natalism (Roberts, 1999). For 19th century states seeking to consolidate power, the womb was an ideal space to regulate the health of the population, protect the ‘survival’ of the nation, and monitor the ‘integrity of the race’ (Miller, 2007: 101). In France, for instance, the criminalisation of abortion in the late 19th century was explicitly tied to fears about population decline and ‘race suicide’ (Ibid. 18–9). Even in the aforementioned example of Romanian pro-natalism, ethnic Hungarians living in Romania had more access to contraception and faced less pressure than ethnic Romanians to meet birth rate ‘targets’ (Kligman, 1998).

Although the language of ‘race suicide’ is largely absent, these same anxieties about race, population and reproduction are still animating anti-abortion conversations around the world today (see e.g. Bialasiewicz 2006; King 2002). Recent geographical work on population and demography has noted the cyclical nature of anxieties about fertility and demographic decline – as well as their racialized nature – but have failed to situate abortion regulation in this context (see Tyner 2013; (Robbins and Smith, 2017)). Fertility is subject to interventionist management and control by state, medical and social institutions and abortion regulation continues to be a fundamental component of state population strategies. Love, sex, marriage, babies: for groups making territorial claims based on identity, these intimate aspects of life are geopolitical problems (Smith 2020). This is evident in the racialized pro-natalism of Israel (King, 2002; Steinfeld, 2015), Ireland (Calkin, 2019a, 2019b; Fletcher, 2005; Fletcher, 2001), and Poland (Mishtal, 2015). Politicians in white settler states like Australia and the US frequently invoke fertility and abortion to stoke fears about the ‘replacement’ of white populations with non-white ones. Notable examples abound. In 2017, white supremacists marching in Charlottesville, Virginia, USA made headlines around the world when they chanted about how they refused to be ‘replaced’ by ‘Jews’ (Feola, 2020). In 2006, an Australian parliamentarian warned her colleagues that white Australians were ‘almost aborting ourselves out of existence’ (Millar, 2017: 253). Where populist and illiberal politics are on the rise, opposition to abortion forms a key plan of an ‘anti-gender’ agenda, through which nationalists promise to stop the encroachment of pro-abortion and pro-LGBT policies from abroad (Korolczuk and Graff 2018). Abortion’s spatiality is both literal and metaphorical: the uneven landscape of safe and legal abortion generates extensive domestic and international travel (see the section ‘abortion (im)mobilities’). However, this availability – or unavailability – of legal abortion in particular places is shaped by its position in wider visions of nation, state, race and family.

### 3 Abortion regulation and medical control

Second, we turn to the spatial mechanisms of surveillance and control that operate in a doctor-patient relationship around abortion. The medical establishment led the earliest and most influential campaign to criminalise abortion. For example, in the USA, the American Medical Association passed a resolution condemning abortion in 1859 and launched the first campaigns to generate public opposition to abortion to the widespread practice (Petchesky, 1984). Medicalizing abortion and investing doctors with the authority to determine when abortion was medically necessary, was also tied to efforts to elevate the status of the profession in class terms, and to distinguish doctors from other forms of faith healers, midwives and nurses (Mohr, 1979; Luker, 1985). Physicians’ groups had been the most vocal advocates of criminalization during the 19th century, and they were also some of the first and most prominent advocates for legalization from the 1950s onwards (Reagan, 1998). By and large, states that criminalized abortion in the late 19^th^ century maintained exceptions for ‘therapeutic’ abortions needed to save the lives of pregnant women, although doctors differed widely in the way that they interpreted the meaning of therapeutic abortion (Luker, 1985).

Abortion bans had never been fully effective or fully enforceable, and abortion continued to be available for women of means. The illegal abortion trade appeared in the public imagination as a dark, dirty and underground scourge: ‘backstreet’ abortions created a spatial imaginary of illegitimate abortion outside of medical authorization and facilities (Millar, 2017). If legal reform of abortion provision sought to bring it out of the ‘backstreet’, it did so by placing it in the clinic and vesting decision-making power over abortion in the hands of medical professionals and by extension the government. The persistence of medical gatekeeping over abortion is still pervasive in the continuing patterns of geographical inequality in abortion access: even countries with permissive laws or decriminalised abortion continue to see enormous disparities between different states and regions, leading to long-distance travel to access abortion (Sethna and Davis, 2019; Sethna and Doull, 2012; De Zordo et al., 2016).

Abortion laws are often vague and by default they delegate physicians and abortion providers with interpreting them (Erdman and Johnson, 2018). By extension, laws that criminalize abortion also delegate surveillance authority and responsibility to physicians. This is evident in the way that medical spaces can function as sites of criminalization for patients who are suspected of attempting to end their pregnancies with medication abortion or any number of abortifacients (Goodwin, 2020). To draw from a further American example: research by Paltrow and Flavin (2013) demonstrated the way that geography, race, class and power relations of particular places shape the practice of reproductive medicine and the exercise of medical authority. Paltrow and Flavin identified pregnancy-related criminal prosecutions in 44 states but noted that South Carolina accounts for 23% of the nationwide total. Of the 93 cases they identified in South Carolina, 30 had been filed by a single hospital (Paltrow and Flavin, 2013; Oberman, 2018). Criminalisation amounts to a spatial lottery: what is treated as a miscarriage in one hospital or state might be treated as a criminal case in a neighbouring hospital or just over the state border. Nor are these inequalities an accident: they are the product of longer historical legacies where the reproductive lives of racialized women are pathologized, surveilled and criminalized (Roberts, 1999; Solinger, 2007).

The clinic is a classic site for the study of power relations. Clinicians can exercise power over abortion-seekers in ways that stigmatize, harm or criminalise them. They can also act as essential allies and advocates for abortion, creating and maintaining the physical spaces where safe abortions take place. Geographers and sociologists have illustrated the steps that abortion providers take to create clinic spaces that are safe and accessible. In places like the US with highly mobilised anti-abortion protestors, this can mean building clinic spaces with special consideration for patient anonymity or establishing networks of volunteers who physically escort abortion seekers through hostile crowds (Brown, 2006; Cohen and Joffe, 2020). In border zones where abortion laws differ significantly on either side, abortion providers locate themselves and provide special language services, outreach services and funding mechanisms (Mishtal, 2015; Sethna and Davis, 2019). At different scales, abortion spaces are made and contested: at the national scale, by law and regulation; at the regional scale, by cross-border availability; at the clinical scale, by the prevailing medical culture and the actions of abortion providers; at the community scale, by support groups and funds. Crucially, these scales are not discrete, and the following section explores mobility as a lens through which to examine the geographies of abortion.

## IV Abortion (im)mobilities

The third theme that shows that abortion is profoundly geographical and should be placed at the centre of geographical analyses is mobility. Here we focus on the power and politics of abortion mobilities, defined as ‘the movement and fixity of people and things that shape abortion access’ (Freeman 2020, 896). Although the relationship between health and medical inequalities, gender and mobility has been vastly understudied, the burgeoning field of reproductive mobilities is beginning to address this (Schurr, 2018). As Mimi Sheller (2020) has argued, bringing mobilities and reproduction scholarship together is fruitful on both sides; each can be used to inform, foster and give new insights into the other, leading Frohlick et al. (2019) to use the term ‘reproductive + mobilities’. Abortion is starting to be studied within this framework (see Murray and Khan, 2020), but most scholarship has still focussed overwhelmingly on *procreative* reproductive mobilities. A geography of abortion can act as a bridge here by working at the axis of mobility and reproduction in a multi-scalar way that encompasses bodies, transport, spaces of healthcare, nations and the planet. In this section on abortion and mobility we focus on barriers that prevent mobility, the privilege of immobility, and the role of technology in shaping (im)mobilities.

### 1 Barriers to mobility

Mobility is fundamentally shaped by power and this creates inequalities of movement (Skeggs, 2013). It is therefore essential that a geography of abortion explores the power relations that affect both movement and stasis (Hannam et al., 2006). Some people, regardless of the legal status of abortion where they are or how accessible it is, are able to travel internationally or domestically for a safe abortion. Abortion travel has occurred on a global scale since the 1960s (Sethna and Doull, 2012), but this option is stratified by class and economic privilege. Factors including arranging childcare, finding accommodation, booking and paying for transport, and taking time off from work, all make travelling for abortions burdensome and even impossible (Doran and Hornibrook, 2016). These barriers fall unevenly, and it is young women, indigenous women, rural women and women on low incomes who are disproportionately affected (Doran and Nancarrow, 2015; Silva and McNeill, 2008). Some people have a greater capability for mobility than others (Sheller, 2018).

Research has also shown that when people do travel, their experiences of mobility are not equal. Those who rely on public transport may encounter limited services that do not offer suitable routes (Brown, 2019; Gomez, 2016) or are forced to travel on private, costly transport such as taxis or ride-sharing apps (Marty, 2019). A significant issue with the current scholarship on abortion mobilities is that it is almost entirely focussed on North America, Europe and Australasia and primarily in locations where abortion is legally accessible (Barr-Walker et al., 2019). This geographical bias in scholarship means we know little about barriers to mobility in the global South. Across Africa, for example, abortion services are predominantly located in urban areas and are inaccessible for those in rural areas or even in many urban areas, and these issues are exacerbated by the absence of transportation (Hord et al., 2006). As a result, those seeking an abortion are unable to travel, the trip would take several days, or it would be prohibitively costly.

Geographers should be well placed to analyse these barriers but much of the most spatially attuned abortion access scholarship has been conducted by legal scholars. Statz and Pruitt (2019) for instance have challenged assumptions about the ‘emptiness’ of distance by demonstrating the obstructions to abortion access in Texas and how rural distance is material, legal and gendered. In questioning ‘urbanormativity’, legal scholars have shown how law is spatially variegated, placing particular burdens on rural women with regards to abortion access (Pruitt and Vanegas, 2015). Huddleston (2016), moreover, has explored the discriminatory logic of internal border checkpoints in Texas that force undocumented migrants to risk deportation if they travel for a safe, legal abortion. Critical, geographical work on barriers to abortion access is happening, therefore, but very often in legal studies rather than in geography.

### 2 The privilege of immobility

Scholarship on abortion travel has predominantly focussed on the ability to move, yet stasis can be the apex of privilege. In jurisdictions where abortion is illegal, wealthier women are more likely to be able to pay the steep fees for a doctor who will perform the procedure clandestinely but safely, and this line of privilege has persisted across space and time. Across the world, for centuries, women with means have been able to ensure their safety, privacy and stasis. The privilege of immobility is, however, being offered to a wider range of abortion-seekers through the changing geographies of abortion pills. Pills are mobile, and transnational medication activists move them in creative ways, like the ship campaigns of Women on (Calkin, 2020; Marston, 2000) Waves or the cross-border air/land/sea routes of activists across Ireland (Calkin, 2019a, 2019b). As Sheller and Urry (2006) have argued, the mobility turn needs to account for how materials move, not just how people move. The availability of the abortion medications mifepristone and misoprostol varies greatly across jurisdictions and in some locations they are obtained in one jurisdiction and transported back home by moving bodies where they are taken. For example, misoprostol is more easily obtained in Peru than in Chile and so women have travelled from Chile to Peru to buy the medication and then smuggle it back into Chile to use it (Freeman, 2017). Yet, the medication is not always transported in person. Postal services are employed by individuals and activists who transport abortion pills transnationally (Sethna and Davis, 2019), making abortion pills ‘readily accessible via a few clicks of a mouse’ (Sheldon, 2016: 90). As technologies emerge to expand the geographical mobility of abortion pills, corresponding restrictions are imposed to limit their reach (Calkin 2022).

The political potential of the mobility of abortion pills has particularly benefited those who have been most affected by barriers to access. This includes those who lack financial resources or are unable to take time off from work or caring responsibilities, but also those whose mobility is most intensely governed by the state. In the Republic of Ireland, for example, migrants must apply for permission to travel abroad which requires time and money (Bloomer et al., 2019; Side, 2016). Moreover, studies of the abortion pill misoprostol on the Thailand–Burma border (Tousaw et al., 2018) and in Peru (Duffy et al., forthcoming) have shown the importance of the transportation of medication to provide access to reproductive healthcare beyond the constraints of the nation-state.

Abortion mobility has been disproportionately studied over abortion immobility (Side, 2020), but it is important to consider the distinctions between voluntary and involuntary immobility. Immobility through insurmountable barriers to abortion access is highly oppressive with significant health and emotional consequences whereas voluntary immobility can mean safer abortions without the need for travel. The mobility of abortion pills can therefore be seen as ‘emancipatory’ as it can help to avoid the often traumatic and humiliating experiences associated with abortion travel (Freeman, 2020). And yet, abortion pills are not emancipatory for all. They are not suitable for later pregnancies and are not appropriate for people with certain health conditions or an ectopic pregnancy (Kapp and Lohr, 2020). This all means that abortion pills are an imperfect mobile object but, in many cases, when abortion pills move instead of people, those seeking abortions can manage their own fertility on their own terms.

### 3 Technologies of (im)mobility

The politics of abortion (im)mobility has been strongly shaped by technology. The mobility turn has been attuned to the movement of ideas as well as people and things (Cresswell, 2016), and one technology that has affected the geographies of abortion is safe-abortion hotlines. The aforementioned transportation of pills relies on two things: the knowledge that such mobility exists, and knowledge of how to safely administer the pills. Although much information on the safe and effective use of misoprostol is available, those seeking abortions often lack access to this information or encounter inaccurate and potentially harmful information (Erdman, 2012; Hyman et al., 2013). Hotlines therefore attend to the failure of governments to provide safe reproductive healthcare by providing accurate information (Jelinska and Yanow, 2018), and are indicative of a wider feminist project of empowering women to take control of their own reproduction outside of medicalized settings. They are particularly prevalent across Latin America and their main advantage as a technology is that they require little infrastructure; the main quality needed is that the operators have the requisite knowledge to support callers (Drovetta, 2015).

There is a mobility and geography to how knowledge of the existence of hotlines is shared that can be traced through space and time. Although people (predominantly women) have long shared information about how to safely procure or administer an abortion (Moore, 2013), the mobility of this knowledge has been transformed by technology. The use of hotlines, and more recently websites, has democratised knowledge and expanded its geography. Rather than needing to know someone with the necessary knowledge, people are now able to access it more anonymously and more safely. This access often occurs in public space with hotline phone numbers plastered, scrawled and graffitied in public restrooms, restaurants, trains and on walls as well as on social media platforms (Bloomer et al., 2019; Erdman, 2011). Hotlines therefore make knowledge about abortion mobile and can lead to improved health outcomes for women in locations where abortion is legally restricted (Gerdts and Hudaya, 2016).

As long as restrictive laws prevent people from accessing safe, legal and local services, mobility is central to the geography of abortion access. Through studying barriers to access, the privilege of immobility, and the role of technology, scholars have explored who gets to access safe(r) abortions and where they occur. Through these three examples, it is possible to see how abortion access cuts across scale; from transnational travel to graffiti on a street corner, the spaces in which abortion is accessed are changing. By focussing directly on abortion, the geographical nature of how women’s bodies are governed and how the movement of people, pills and ideas is allowed, prevented or erased becomes clear.

## V Conclusion

In this paper, we have made a case for a geography of abortion. Although vibrant scholarship on abortion has often had a geographical sensibility, geographers themselves have been late to these discussions and have too often been shy of them. The paper drew attention to the ways in which the conversation around abortion, both academically and otherwise, is changing. The three themed sections in the paper on discourse, spatiality and mobility have shown that abortion has been and continues to be a highly geographical phenomenon. The purpose of the paper’s key themes was to make visible new approaches including linkages through space and time that have been hitherto unseen in academic investigations of the topic.

We conclude by offering three questions to shape future research in this field: What are the limits of legal approaches? How can we further theorise caring relations through abortion and abortion activism? And what are the geographies of knowledge production on abortion?

First, abortion continues to be hotly debated in the legal sphere in terms of the liberalization of abortion in countries such as Argentina and New Zealand but also increasing restrictions in locations such as Poland and Honduras. Rights, then, in a legal sense, are necessary subjects of study but rights are insufficient, as the Reproductive Justice movement and associate scholarship has illustrated (Ross and Solinger, 2017; Rebouché, 2017). A legal right to abortion, for instance, is not meaningful for people without the power and resources to exercise that right. Reproductive Justice scholars have been rightly critical of the mainstream reproductive rights movement, with its focus on abortion (at the expense of other reproductive activities like childbirth and child rearing) and its underdeveloped account of class and economic justice (Luna and Luker, 2013; Nelson, 2003). We therefore argue that it is essential to place abortion scholarship within broader contexts to also address the oppressive racial, historical, economic and sexual inequality structures within which rights can be exercised or not (Gurr, 2011).

Second, feminist theories of care are essential to understand the complexity of this uniquely stigmatized healthcare procedure. This complements geographical foci on emotions and affect (Duffy, Freeman, Rodriguez et al., forthcoming; Calkin and Freeman, 2019), and we encourage future scholarship to situate abortion care within the national, global and historical forms of governance that shape abortion access. Studying the changing geographies of abortion requires us to reckon with its re-spatialisation: moving beyond clinical spaces of care, extending across borders, encompassing new digital platforms and communication systems. Abortion activism and clandestine provision of safe self-managed abortion (where abortion is iillegal or difficult to access) construct alternative structures for realizing abortion care in the absence of legal change (see Drovetta, 2015, for example). We call for greater engagement between geographical scholarship on abortion and scholarship on caring relations, particularly those that work outside of formal institutions and market relations.

Third, the geography of abortion geography scholarship and reproduction scholarship more broadly has been heavily skewed toward the global North both in terms of who conducts research and who is the research’s ‘subject’. This leads to the flattening of reproductive lives and experiences in the global South and reproduces particular reproductive geographies (Bagelman and Gitome, 2020). In this paper, we have shown the geographical linkages across space and time that are so crucial to our understanding of abortion, and which also offer so much potential for the study of related concepts such as citizenship and gender and we call for a greater critique of which abortion geographies and experiences are studied and the funding, institutional and political contexts that dictate who can conduct this research.

Coda: As we edited this article in summer 2022, the US Supreme Court overruled 50 years of precedent, declaring that there is no constitutional right to abortion in the USA. Within minutes of the decision, American states moved to enact abortion bans; in total, 26 out of the 50 states are expected to pass abortion bans. Suddenly, detailed discussions of abortion geography ran on the front pages of major newspapers around the world. Although abortion access across the states had been profoundly uneven – six states had only one abortion clinic each – the removal of the constitutional abortion right created a complex patchwork. Ideas like ‘abortion deserts’ – regions without a provider for hundreds of miles – entered public discussion, along with the abortion ‘oasis – an abortion-friendly state encircled by abortion-hostile states. Overnight, the fundamentally geographical nature of abortion became apparent to the most casual observer. The average American will need to travel 250 miles further to access their nearest clinic, provided they can overcome the complex obstacles that effectively make long-distance abortion-travel a class privilege (Myers et al., 2019; Statz and Pruitt, 2019). Abortion’s geography, we have argued, accounts for but extends well beyond the question of distance between abortion seeker and abortion provider. It also enrols the geographical discourses that structure gender relations, the forms of reproductive governance that regulate pregnancy, childbirth, child-rearing, and society-state relations, and complex forms of human and non-human mobility that shape people’s relationship to reproductive autonomy. At a moment of disruption, transformation and outrage, geographical scholarship on abortion and reproductive justice is more urgent than ever.
